# Virtual Versus In-person Grand Rounds in Orthopaedics: A Framework for Implementation and Participant-reported Outcomes

**DOI:** 10.5435/JAAOSGlobal-D-21-00308

**Published:** 2022-01-19

**Authors:** Gireesh B. Reddy, Marcella Ortega, Seth D. Dodds, Mark D. Brown

**Affiliations:** From the Department of Orthopaedics University of Miami Miller School of Medicine, Miami, FL.

## Abstract

**Introduction:**

Grand rounds have been weekly gatherings at academic orthopaedic surgery programs across the country for decades. During the 50th year of grand rounds at our institution, the COVID-19 pandemic prompted the transformation of this in-person forum into a virtual setting. The purpose of this study was to detail this initiative and to report survey data providing participant-reported perceptions and satisfaction of virtual versus in-person grand rounds.

**Materials and Methods:**

Once in-person meetings were discontinued, virtual grand rounds commenced using the Zoom video application. At the conclusion of the 2020 to 2021 academic year, a 30-item online survey was sent to all residents, faculty, and visiting faculty to assess their perspective and satisfaction. A five-point Likert scale ranging from 1 to 5, with 5 being extremely effective, was used. A 21-item follow-up survey was sent to all speakers as well.

**Results:**

Thirty-six virtual grand rounds were successfully hosted. The response rate for the survey was excellent—80 of 86 (93.0%) surveys returned completed. Respondents found that virtual grand rounds were more convenient to attend, were more convenient to obtain Continuing Medical Education, and were more satisfied with virtual grand rounds. Respondents reported that in-person grand rounds were more effective for stimulating social collegiality and networking. Speakers found that virtual grand rounds were more effective for uploading the presentation and overall convenience, whereas they were less effective at retaining audience attention and receiving audience feedback. Improved faculty attendance after the switch to virtual grand rounds was also noted.

**Conclusion:**

This study found that respondents across all groups appreciated the convenience of attending and obtaining Continuing Medical Educations at virtual grand rounds while also noting the merits of in-person grand rounds for promoting peer interaction, camaraderie, and departmental identity. All respondents strongly recommended continuation of this program in a hybrid format. Virtual orthopaedic grand rounds are viable, readily implemented and demonstrate improved participant satisfaction.

Grand rounds' format lectures have been a cornerstone of medical education and a stalwart presence at many academic medical and surgical departments across the country. Since their inception in the late 19th century by Sir William Osler, they have transformed from initial bedside rounds and discussions of patients to large, symposium style presentations in auditoriums.^1-3^ From changing focus on individual interesting or perplexing cases to the broader exploration and characterization of disease and of surgical techniques, grand rounds' lectures adapted with the growing breadth and depth of medicine.^4^ Technologically, grand rounds have advanced as well from physically examining a particular patient and offering suggestions for care to all-encompassing PowerPoint style presentations given by expert clinicians and researchers.^2,3^

Our institution's Orthopaedic Grand Rounds have been presented every Thursday morning during the academic season since 1970. Historically, Grand Rounds at our institution were started by Dr. Augusto Sarmiento in 1970 and taken over by Dr. Newton McCullough in 1979. Both past presidents of the American Academy of Orthopaedics were successful in inviting guest speakers from all over the world and facilitating discourse. From 1986 until present, grand rounds are managed by one of the authors who built on their successes. Current grand rounds are an endowed, Continuing Medical Education (CME)-accredited, and CME-archived lecture series. During the 50th year of presentations, early in 2020, the COVID-19 pandemic forced us to adapt to a new paradigm of delivering grand rounds. We implemented an online venue to replace the live presentations, taking care to retain and continue the quality and tradition of the past. Careful attention to conflict of interest, quality of presentations, pertinence, and interaction of participants was considered in the transition to the online venue.

We report the methodology and our institutional experience in transitioning from in-person to virtual ground rounds and data comparing both in-person grand rounds from April 2019 to March 2020 and virtual grand rounds from April 2020 to March 2021. This comparison regarding implementation, participation, attendee perception and satisfaction, overall cost-effectiveness, and planning for the future is presented.

## Methods

Perhaps one of the initial critical factors in transitioning to virtual grand rounds was the selection of a robust platform to host the grand rounds' presentations and interactions. Internet-based conferencing tools, such as Microsoft Teams, Google Chat, and Skype, have facilitated communication and collaboration in academia and business for past several years. The variety of the online videoconferencing applications has been previously described.^5-7^ Although a multitude of viable platforms exist, only Microsoft Teams and Zoom, a relative newcomer in the space, were licensed by our institution. Given that many of our peer institutions were incorporating Zoom (Zoom Video Communications), we chose similarly. Because many have found, Zoom's functionality, simple user interface, and framework that easily facilitates speakers and audiences further solidified its role as a key component of the transition to virtual grand rounds.

After the technological platform was selected, we began working directly with the CME office at our institution to facilitate with the transition of our department from conducting in-person grand rounds' sessions to fully virtual. Between 2019 and 2020, we had in-person grand rounds organized so that those who attended in person were documented and received 1 CME credit hour per session. All university faculty, residents, and fellows were responsible for signing in and gaining CME credit for each session. In-person grand rounds originally had an alphanumeric code or QR code sign-in that could be scanned and sent to the CME office to document attendance for credits. This process was converted to an online platform for virtual grand rounds. In coordination with the CME office, we developed an online CME tracker to document CME credits for participants. Grand rounds were recorded and made available online for participants to view and obtain CME credits up to a later date. Presentations were also archived and assessable through the department website.

At the conclusion of the 2020 to 2021 year, a 30-item online survey was created using Google Forms and distributed to all residents and faculty who were present for both years, as well as all visiting faculty to assess their perspectives on the in-person versus virtual grand rounds. A five-point Likert scale ranging from 1 to 5, with 5 being extremely effective, was used. A 21-item follow-up survey was sent to all speakers as well. Statistical analyses comparing responses for in-person and virtual grand rounds were done using JMP Pro (Cary, NC). Individual variables from the online intervention assessment data were averaged. Likert data were considered both to be continuous and parametric,^8^ and pooled two-sampled Student *t*-tests were conducted to compare the results between perceptions regarding in-person and virtual grand rounds. Likert data from the online survey were then visualized as a stacked, Gantt bar chart using Supplemental Table 2, http://links.lww.com/JG9/A182.

Regarding attendance, residents were required to sign in and percentage attendance was tracked using their sign-in data between in-person and virtual grand rounds. For faculty, no specific sign-in was required; therefore, percentage attendance for each faculty was calculated using a number of sessions where CME credits were registered divided by the total number of in-person or virtual grand rounds' sessions. Matched pairs Student *t*-tests were conducted between in-person and virtual grand rounds' percentage attendance to ascertain statistical significance. A value of *P* < 0.05 was considered statistically significant for all instruments.

## Results

Throughout the 2020 to 2021 academic year, we successfully hosted 36 virtual grand rounds. The response rate for the survey was 80 of 86 (93.0%) surveys returned completed, with 100% of the residents and 94.3% of the faculty responding (Table 1). Presenters included 13 of 27 residents (48.1%), 17 of 33 faculty (51.5%), and 20 of 20 visiting faculty (100%) (Supplemental Table 2, http://links.lww.com/JG9/A182). Residents and faculty found that virtual versus in-person grand rounds were more convenient to attend (4.655 versus 2.276, *P* < 0.0001 for residents, 4.939 versus 2.613, *P* < 0.0001 for faculty); more convenient to obtain CME (4.292 versus 3.12, *P* < 0.0001 for residents, 4.667 versus 3.355, *P* < 0.0001 for faculty); and were overall more satisfied with virtual grand rounds (4.276 versus 3.655, *P* = 0.0073 for residents, 4.576 versus 3.849, *P* < 0.0001 for faculty) (Supplemental Table 1, http://links.lww.com/JG9/A182). In addition, residents reported that the virtual format was more effective in organizing collaborative grand rounds (4.222 versus 3.148, *P* < 0.0001). By contrast, residents and faculty reported that in-person grand rounds were more effective in facilitating collaboration between presenters and participants (4.104 versus 3.31, *P* = 0.0054 for residents, 4.323 versus 3.545, *P* = 0.0023 for faculty), and social collegiality and networking (4.172 versus 2.655, *P* < 0.0001 for residents, 4.065 versus 3.03, *P* = 0.0009 for faculty) (Supplemental Table 1, http://links.lww.com/JG9/A182). For visiting faculty, virtual grand rounds were markedly more convenient to attend (4.563 versus 3.545, *P* = 0.0115) (Supplemental Table 1, http://links.lww.com/JG9/A182). No notable differences were found for visiting faculty in overall satisfaction, and no notable differences were found for all respondents in providing medical/research updates and in educating the audience. The distribution and visualization of the Likert data along with the mean score for each survey item across each respondent group are shown in Supplemental Figure 1, http://links.lww.com/JG9/A181. When asked what delivery format participants would prefer in the future, respondents largely preferred a hybrid approach. This preference was consistent across residents (19/27, 70.4%), faculty (27/33, 81.8%), and visiting faculty (15/20, 75%) (Table 2).

**Table 1:** Baseline Characteristics of Respondents

Respondent Category	Number Responded	Number Sent	Response Rate
Residents	27	27	100%
Faculty	33	35	94.3%
Visiting faculty	20	24	83.3%
Total	80	86	93.0%

**Table 2:** Future Format Preferences by Respondent Category

Respondent Category	Hybrid	In-Person	Virtual	Total
Residents	19	2	6	27
Faculty	27	1	5	33
Visiting faculty	15	4	1	20
Total	61 (76.25%)	7 (8.875)	12 (15%)	80

Speakers preferred the virtual grand rounds' format for uploading/organizing their presentations (4.3215 versus 3.7353, *P* = 0.0093) and for overall convenience (4.5475 versus 3.500, *P* < 0.0001). Conversely, speakers noted that in-person grand rounds were more effective for retaining audience attention (4.1177 versus 3.3514, *P* = 0.0024) and receiving audience feedback (4.0294 versus 3.4879, *P* = 0.0445) (Supplemental Table 3, http://links.lww.com/JG9/A182). There were no notable differences in preparing or delivering the presentation, ease of presenting, or overall speaker satisfaction between in-person and virtual grand rounds (Supplemental Table 3, http://links.lww.com/JG9/A182). Percentage attendance almost doubled among faculty for virtual grand rounds versus in-person grand rounds (57.06% versus 29.12%, *P* = 0.0004). No notable difference was noted for resident percentage attendance between virtual and in-person grand rounds (Table 3). Cost of virtual grand rounds was dramatically less expensive overall compared with in-person grand rounds ($2500 versus $25,468.85) (Table 4).

**Table 3:** Overall Grand Rounds' Attendance by Year

Group	In-person % Attendance Mean	Virtual % Attendance Mean	% Increase	*P*
Faculty	29.12%	57.06%	95.4%	***0.0004***
Residents	79.99%	82.11%	2.65%	0.5556

*P <* 0.05 using a matched pairs Student *t*-test

**Table 4:** Overall Grand Rounds' Cost per Year by Format

Line Items	In-person Cost, $	Virtual Cost, $
Facility fees	$21,600	$0
Speaker travel expenses	$1368.85	$0
CME fees	$2500	$2500
Total	$25,468.85	$2500

CME = Continuing Medical Education

## Discussion

Because of the COVID-19 pandemic and the suspension of in-person gatherings and meetings, we initiated a change from our in-person grand rounds to weekly virtual grand rounds. We report our experience with this transition and participants' perspectives on in-person and virtual grand rounds. Although web-based live communications and team meetings have been mainstays in large corporations and technology companies,^9,10,11,12^ the adoption of similar methods of communication and knowledge exchange has been slow in medicine but has been accelerated dramatically in the midst of the COVID-19 pandemic.^5,13-15^ Similar to what has been described in initiatives across other medical specialties,^16-18^ we found that respondents across all groups appreciated the convenience of attending and the convenience of obtaining CMEs at virtual grand rounds. Virtual grand rounds have allowed faculty and residents who normally have clinic or cases scheduled off-site or soon after grand rounds conclude to easily join in either together at an off-site location or individually from their personal electronic devices. This corresponded to a 95.4% increase in the attendance rate of faculty (Table 3), likely due to faculty being able to join virtually while doing other work. Although a minor increase was noted in resident attendance (82.11% versus 79.99%), this was likely due to grand rounds being mandatory for residents even before the change to virtual. The slight increase in attendance can be attributed to attendance being more consistently recorded by our program coordinators instead of relying on residents to sign in. In a survey study of 87 physicians across several hospitals in Canada, grand rounds were felt to be the primary form of acquiring CME within the hospital and that the most important aspect of grand rounds was in providing updates in research, diagnosis, and management.^19^ Our study demonstrates that respondents found that virtual grand rounds were noninferior to in-person grand rounds in achieving major learning objectives and were a far more convenient and accessible delivery format.

Residents also perceived that the virtual format was more effective for collaborative grand rounds, that is, within and between departments and with other institutions. This sentiment was also present among the faculty and was nearly statistically significant (*P* = 0.0511). Intradepartmental and interdepartmental collaboration is critical to further the combined goals, interests, and learning of individuals and departments as a whole. Often, some of the best attended and best received grand rounds are instances where a speaker encompasses multiple disciplines to engage the audience. Taken together, residents and faculty were more satisfied with virtual grand rounds. Several studies in the past have shown high overall participant satisfaction with virtual learning and virtual grand rounds^20-22^; however, few, if any, offer a direct comparison between in-person and virtual grand rounds. Although visiting faculty satisfaction was not statistically significant between the two formats, this was likely due to not all presenting at both formats at our institution, therefore mitigating the effect of the change. Virtual grand rounds also impart notable cost benefits to departments. More often than not, there are no associated direct costs only and indirect costs would be the video teleconferencing software licensing. This generally is included under a university or health systems' information technology infrastructure. Only a few individuals in the department would need access to “pro” or “upgraded” licenses of teleconference software, such as Zoom, to have conferences that have expanded audience member and meeting duration limits. In-person grand rounds have tangible, direct, and indirect costs associated with them. Direct costs include expenditures such as renting the physical meeting space weekly, refreshments, travel, lodging, dining accommodations for the speaker, and gifts of appreciation for the speaker. The cost benefit of the change to virtual grand rounds is reflected in a dramatic decrease in the yearly costs, $25,468.85 for in-person versus $2500 for virtual (Table 4). A financial analysis of in-person versus virtual grand rounds at a peer institution demonstrated similar results with a net cost savings of $388.82/month with virtual grand rounds.^23^

There are, however, tangible disadvantages associated with virtual grand rounds. Faculty and residents found that collaboration and interaction between the presenter and participants was less effective in a virtual setting. This sentiment was also reflected in speaker feedback: Speakers found it more difficult to retain audience attention and to receive any feedback on how the presentation was received. Often, even with the best delivered and most interesting virtual presentations, there was hesitancy between the conclusion of the talk and any questions or comments from the audience. In-person reception can often be measured by the interaction between the presenter and participants during the talk, immediately after the talk during formal or informal question and answer sessions, and in any planned events afterward. In virtual grand rounds, the only opportunity for conversation may be during the question-and-answer session. Although a question-and-answer segment can be a surrogate for reception, it may be more dependent on how interesting or controversial the topic was.

Ongoing research on electronic learning during the COVID-19 pandemic from elementary education to the CME of allied health professionals has similar findings, with students or participants losing engagement and interaction while simultaneously becoming more isolated learners.^24,25^ The concept of Zoom fatigue has also been described recently.^26^ In essence, videoconferencing necessitates us to focus more deliberately to avoid loss of information; however, constant interruptions and opportunities for distraction can be detrimental to our productivity and energy.^26^ Scheduled breaks and reducing multitasking distractions can be key tools in mitigating lost productivity and decreasing learner strain. Some aspect of a delivery is also lost in the virtual format—the intimacy of a presenter holding individual audience member's attention, using hand gestures and movement in the presentation space to one's advantage, and most poignantly, the effect of anecdotes and humor are lost without a responsive and expressive audience. The only indication that a virtual grand rounds went well may be direct feedback from a few individuals. As Plancher et al^5^ opine on the changing face of orthopaedic education,^5^ is there some degradation of the educational experience if we are unable to fully disconnect from the tasks of the morning, either personal or at work, to fully pay attention to the presentation? Although the authors' points more specifically focus on annual meetings and teaching conferences, the same question can be asked of grand rounds.

In-person grand rounds also serve an important purpose in playing a weekly role in building departmental camaraderie and identity between all participants. Orthopaedic surgery departments can be large, and faculty and residents can be dispersed across multiple sites. Grand rounds are an opportunity for faculty and residents to literally and proverbially look around the room and take stock of their department, its culture and structure, and their role and contribution to it. Standaert et al^11^ wrote in an analysis of pandemic business meetings, “business meetings function as a key venue to create, negotiate, and disseminate organizational culture, and serve as a powerful social symbol, making the organization and its structure visible and apparent to its members.” Interestingly, and in contrast to other studies, Stein et al^27^ found in a study evaluating a national virtual fracture conference initiative, participants found the quality of their interpersonal interactions to be superior to that of their home institutions. As we continue to incorporate virtual education in an evolving pandemic, it may be crucial to evaluate the individual components of interpersonal interactions, professional camaraderie, and organizational identity to create technological applications and meeting formats that best recapitulate those critical aspects.

This study is not without limitations. This was a single-center study over a cumulative period of 2 years. The results are most accurate when considered in the context of a moderately sized academic institution such as our own. Surveys can be subject to sampling bias. We sent questionnaires to all residents and faculty who were present for both in-person and virtual grand rounds. Notably, this meant that Post-graduate year-1 (PGY-1) residents did not meet the inclusion criteria for the study. It can be inferred that junior residents may not have felt that virtual grand rounds were as protected as in-person grand rounds, thereby diluting their educational benefit. In a similar survey study of virtual lectures for a general surgery residency program during the pandemic, Nagaraj et al^28^ found that although most residents in their program noted an increased ability to attend virtual conferences and a majority reported to prefer continuation of virtual conferences, PGY-1 residents reported the changes negatively and PGY-2 residents felt neutral in comparison to positive responses from other postgraduate years. Our resident results for virtual grand rounds may be more positively skewed because of this exclusion. Response bias is also commonly cited with online surveys. Through in-person and weekly reminders, we were able to achieve an overall excellent response rate with a cumulative response rate of 93.0%.

Moving forward into the 2021 to 2022 academic year, we are incorporating more hybrid sessions into the grand rounds' schedule. These will be for select visiting faculty in-person visits and when coronavirus numbers are at a lower threshold. In addition, we hope to begin virtual case discussions with visiting faculty as a part of weekly resident didactics, providing an opportunity and environment for visiting faculty and resident interaction.

## Conclusions

Overall, during the 2020 to 2021 academic year, we were able to successfully transition grand rounds from a long-standing in-person conference to a virtual setting. Using an end-of-year online survey, we found that attendees strongly preferred virtual grand rounds for its convenience and flexibility but did note the merits of in-person grand rounds for promoting peer interaction and camaraderie. Respondents strongly recommended continuation of this program in a hybrid format for the future. Because COVID-19 continues to affect orthopaedic departments nationally, most recently at the time of this writing with the Delta variant surge resulting in decreased elective case volumes and limited in-person gatherings, virtual grand rounds has become a prudent choice. We plan to incorporate some of the strongest attributes of the in-person experience with the convenience of virtual grand rounds by offering select hybrid conferences this coming grand rounds' season.
